# Beirut blast: creating our expectations through heartbreak

**DOI:** 10.2144/fsoa-2020-0171

**Published:** 2020-11-09

**Authors:** Wael Abdallah, Khalil Khalil, Bernard Najib, Nadine Kassis, David Atallah

**Affiliations:** 1 Department of Gynecology & Obstetrics, Hôtel-Dieu de France University Hospital, Saint Joseph University, Beirut, Lebanon

**Keywords:** ammonium nitrate, Beirut explosion, carcinogenesis, fertility, pregnancy, women’s health

A terrible explosion of ammonium nitrate occurred on 4 August 2020, killing 270 people and injuring more than 6000, in the Lebanese capital: Beirut. This disaster stole the show from all that is happening in the country like revolt, corona and bankruptcy.

Officially, a fireworks explosion had detonated nearly 3000 tons of ammonium nitrate stored in one of the port’s warehouses producing a huge blast [1]. “The most dangerous thing is that no one knows what exploded besides ammonium nitrate and how toxic the dust is,” reclaimed Greenpeace Middle East and North Africa program manager in Beirut [2]. Lebanon’s health minister called for an emergency evacuation to all the regions near the blast because of the hazardous and toxic chemicals produced by the incident.

On its own, ammonium nitrate is usually used as a fertilizer, but it is also an essential compound in the production of industrial explosives [3]. It is not combustible but has a fire-promoting effect due to its positive oxygen balance +20%. It enables the content of oxygen in excess. For this reason, ammonium nitrate is the essential oxidizing salt in multiple types of explosives [4].

An explosion involving ammonium nitrate produces toxic ammonia (NH_3_), nitrous oxide (N_2_O) and nitric oxide (NO) gases [5,6]. The nitric oxide reacts rapidly with oxygen to form the toxic nitrogen dioxide (NO_2_) gas, which has a reddish-brown color. The nitrogen dioxide was probably responsible for the color of the cloud observed in Beirut after the explosion. Inhalation of nitric oxide and nitrogen dioxide causes acute respiratory distress syndrome, diffuse distal airway inflammation, pulmonary edema and methemoglobinemia [7,8].

The department of Gynecology and Obstetrics at Hotel Dieu de France Hospital was interested in the hazards and dangers of ammonium nitrate on women’s health as it was the only hospital in the blast region (eastern Beirut and Achrafieh) receiving casualties and treating wounded patients. All the other facilities were destroyed.

The United States Environmental Protection Agency (EPA), the International Agency for Research on Cancer (IARC), and the Department of Health and Human Services (DHHS) (NTP) did not classify ammonia as a carcinogenic agent [9]. One case report described a patient who developed epidermal cancer of nasal septum 6 months after accidental contact with an ammonia refrigerator [10]. The carcinogenic effect of ammonia after inhalation has not been evaluated in animals and humans. According to the New Jersey department of health, no tests proved that ammonium nitrate exposure caused cancer in animals. Moreover, Kim *et al.* concluded that ammonium nitrate did not induce chromosomal aberrations after evaluating its genetic toxicity in Chinese hamster ovary cells [11]. Even though some studies have shown that a higher oral intake of nitrate can increase the risk of ovarian cancer among women [12], there is no data available for the ammonium’s inhalation nitrate’s carcinogenic impact.

We did not find a scientific correlation between ammonium nitrate and its teratogenicity and toxicity on reproduction on reviewing the literature. However, a recent study showed that the inhalation of ammonium nitrate adversely affects sperm quality. Tyrosine phosphorylation and protein kinase A (PKA) activity were altered by ammonium nitrate regardless of capacitation. Inhalation of ammonium nitrate may reduce sperm motility, capacitation status and motion kinematics, via unusual tyrosine phosphorylation by abnormal PKA activity [13].

Moreover, ammonium nitrate can affect pregnancy. The exposure to nitrate (NO_3_-) during the second trimester of pregnancy is associated with an increase in gestational diabetes mellitus (GDM) [14]. Yu *et al.* reported an increase in nitrate exposure in the second trimester is associated with an increased GDM risk (OR: 1.13; 95% CI: 1.02–1.24). Furthermore, Robledo *et al.* supported that the nitrate increased the risk GDM during the first trimester (OR: 1.05; 95% CI: 1.02–1.09) [15]. Ammonium nitrate affects oxidative stress, endothelial function and inflammation, resulting in insulin resistance and then diabetes mellitus [16].

On the other hand, higher exposure to ammonium (NH_4_+) ions from PM2.5 particulate matter during pregnancy is associated with decreased maternal serum free thyroxin (fT4) levels and reduced birth weight Z score (p < 0.05) [17]. Besides, ammonium nitrate exposure increased the risk of low birth weight (defined as ≥37 weeks and birth weight <2500 g) by 3.1%, especially during the second trimester of the pregnancy [18].

Finally, Basu *et al.* stated that the risk of preterm labor is higher among pregnant women exposed to ammonium nitrate (ammonium 21.2%, nitrate 18.1% [19]. They also reported a greater incidence in Blacks, Asians and older mothers.

## Conclusion

There are insufficient data to evaluate the association between ammonium nitrate and its effect on carcinogenesis, teratogenesis and mutagenesis. The literature review found an association between this toxic agent and fertility among couples, especially in male groups. According to some reports, pregnancies may be affected by some adverse events [16,17,19].

It will be interesting to conduct a prospective study comparing second-trimester ultrasounds among pregnant women living near the blast and a comparator group living far. By viewing the adverse embryologic and gestational events, we can understand the toxicity of ammonium nitrate on both victims: the mother and the baby.

To conclude: “Let me tell you who are the children of my Lebanon… They are the ones who are steadily moving toward perfection, beauty and truth. You have your Lebanon, and I have my Lebanon.” - Gebran Khalil Gebran
